# Examining Test Order Appropriateness Using Mean Abnormal Result Rate in Inpatient and Outpatient Care: A Retrospective Cross‐Sectional Study

**DOI:** 10.1002/hsr2.73052

**Published:** 2026-09-22

**Authors:** Parastou Gorovanchi, Parham Panahi, Zahra Farrokhi, Rasoul Hossein Zadeh, Maryam Baradaran‐Binazir, Rasoul Estakhri

**Affiliations:** ^1^ Department of Pathology School of Medicine,Tabriz University of Medical Sciences Tabriz Iran; ^2^ Kazan Federal University Kazan Russian Federation; ^3^ Shahid Beheshti University of Medical Sciences Tehran Iran; ^4^ Student's Study Committee Faculty of Medicine, Mashhad University of Medical Sciences Mashhad Iran; ^5^ Social Determinants of Health Study Center Health Management and Safety Promotion Study Institute, Tabriz University of Medical Sciences Tabriz Iran; ^6^ Department of Pathology Tabriz University of Medical Sciences Tabriz Iran

**Keywords:** abnormal range, mean abnormal result rate, normal range

## Abstract

**Background and Aims:**

Patients may experience more stress and the healthcare system may incur greater expenditures as a result of doctors ordering too many tests. In order to assess the suitability of testing, the Mean Abnormal Result Rate (MARR) metric—defined as the percentage of tests having abnormal results—was created. Data on how the COVID‐19 epidemic changed test ordering patterns and the corresponding MARR are limited, despite the fact that it greatly increased the demand for laboratory testing, including several tests with no evidence to justify their usage. This study aims to examine MARR in the internal medicine and surgical departments, as well as the emergency and outpatient departments, of Sina Hospital in Tabriz, Iran during the COVID‐19 pandemic.

**Methods:**

Laboratory test results from Sina Hospital's internal medicine and surgical departments, as well as the emergency and outpatient departments, were examined in this cross‐sectional study between March 21, 2021, and March 20, 2022.

**Results:**

The emergency department's mean MARR was 30% (95% CI: 29.8%–30.2%) versus 27% (95% CI: 26.9%–27.1%) in the outpatient department; this difference was not statistically significant (*p* = 0.9). MARR showed wide monthly fluctuations, and a time‐series analysis revealed a negative but non‐significant association between monthly test volume and MARR (*p* = 0.114). After the emergence of new viral variants, MARR decreased from a median of 41% (months 1–6) to 27% (months 7–12), with a borderline significant difference (exact *p* = 0.065).

**Conclusion:**

No significant difference in MARR was observed between clinical settings or specialties. Although MARR and test volume exhibited an inverse temporal pattern, the association was not statistically significant after accounting for autocorrelation. The decline in MARR during the variant wave was borderline significant. MARR may serve as a supplementary metric for monitoring test selectivity, but individual‐level data are needed to assess physician experience or clinical appropriateness directly.

## 1 Introduction

Lab testing is a critical component of healthcare, providing valuable assistance to the physician in diagnosing or eliminating several different clinical diagnoses. However, unnecessary tests ultimately contribute to increased costs, additional risks to the patient, and other negative outcomes. Lab test results usually comprise approximately 60%–70% of a patient's overall objective clinical data, and any delays or barriers that prohibit timely notification of those results can adversely affect the patient's treatment and length of time in the hospital [1]. Lab tests also contribute to unnecessary diagnostic testing and, thus, ultimately the overall effectiveness and outcome of the care provided to the patient [2]. Lastly, unnecessary laboratory testing also tends to create increased levels of anxiety among patients, cause iatrogenic anemia, and add to overall unhappiness [1, 3].

The actual average effect of laboratory tests on medical decisions remains largely unknown; however laboratory testing is widespread for both outpatient and inpatient care, and therefore plays a significant role in escalating costs within the healthcare system [4]. While it is critical to focus on optimization of test orders, there are currently no clear recommendations available for determining which tests should be maximized. Clinical practice guidelines are necessary to reduce unnecessary testing, according to several literature reviews [5, 6]. Initial testing of patients also represents a large factor in over‐utilization of services, highlighting the importance of ordering proper tests during the first evaluation of new patients [7].

In the United States and Canada, the Choosing Wisely program has raised awareness about the need to reduce unnecessary lab tests [8]. The initiative has raised awareness of the issue of low‐value and incorrectly ordered lab tests, with the additional benefit of encouraging more efficient use of clinical resources. However, determining which tests may have been ordered inappropriately remains difficult to ascertain.

The COVID‐19 pandemic problem made everything more difficult. Because controlling COVID‐19 required a number of laboratory tests, such as viral identification [9], study of biomarkers linked to disease prediction [10], and monitoring vaccination effectiveness [11] the pandemic put a great deal of demand on laboratory facilities. It was discovered that many of these tests offered little advantages.

Reducing needless testing is greatly aided by clinical practice standards, however these choices are contingent upon the clinical setting. Tools that enable departmental managers to promptly spot unfavorable patterns in test‐ordering procedures among the many tests that are requested annually are thus desperately needed. In order to quantify the relative appropriateness of testing, the mean abnormal result rate (MARR) concept was established [12]. The quotient of the number of abnormal test findings divided by the number of all ordered tests is known as the MARR. Better testing selectivity is indicated by higher MARRs. MARR scores above a 5% benchmark may indicate that tests are being used selectively to patients with a higher pre‐test probability of disease, increasing the likelihood that a positive result is a true positive, according to the statistical definition of a normal range, which is defined as falling within approximately 95% of results in a healthy population [12]. According to this theory, a score close to 5% might be a sign of excessive test use, where it is requested when not necessary. The goal of this research is to examine MARR for the first time during the COVID‐19 pandemic in the internal and surgical departments, as well as the emergency and outpatient departments at Sina Hospital in Tabriz, Iran.

## 2 Method

### 2.1 Study Design and Setting

Laboratory test results from Sina Hospital's internal medicine and surgical departments, as well as the emergency and outpatient departments, were examined in this cross‐sectional study between March 21, 2021, and March 20, 2022. The ethical committee of Tabriz University of Medical Sciences approved the research protocol (Approval No. 70222). Individual informed consent was waived by the ethical committee because this was a retrospective review of previously collected anonymised data. All laboratory tests listed in Table 1 with non‐zero results for healthy patients met the inclusion criteria, with the exception of vitamin level assessments and serology testing for infectious illnesses.

**Table 1:** Laboratory tests included in the analysis.

Alanine transaminase
Albumin
Alkaline phosphatase
Amylase
Aspartate transaminase
Bilirubin—direct
Bilirubin—total
C‐reactive protein
Calcium
Ca‐125
CBC
Creatine kinase
Creatinine
D‐dimer
Ferritin
Iron
Lactate dehydrogenase
Lipase
Magnesium
Phosphate
Potassium
Sedimentation rate
Sodium
Thyroid‐stimulating hormone
Thyroxine free
Total iron‐binding capacity
Triiodothyronine free
Uric acid
Urea

### 2.2 Data Collection and Statistical Analysis

The mean abnormal result rate (MARR) was calculated as: MARR = (number of abnormal test results)/(total number of test results submitted). This analysis examined 30 different hematology and biochemistry laboratory tests (listed in Table 1). Data were retrieved from the Hospital Information System (HIS). Starting from a total dataset of approximately four million records, duplicate records were removed by retaining only the unique patient identifier and the date of laboratory referral. Tests that did not meet the inclusion criteria, as well as repeated requests for the same test on the same day for the same patient, were also excluded. The final analytical dataset comprised 623,051 test results. All analyses were performed using SPSS software (version 20 for Windows; SPSS Inc., Chicago, IL, USA).

For each component of a CBC/blood differential/PT test, abnormality was determined independently; if any component was outside its normal range, the overall test result was considered abnormal. MARR values were calculated for each clinical department (emergency, outpatient, internal medicine, surgery) and for each individual test by averaging the proportion of abnormal results within that group, allowing direct comparisons between settings.

The Shapiro–Wilk test was used to assess the normality of monthly MARR distributions. Because the data were not normally distributed (*p* < 0.05), the Mann–Whitney U test (two‐tailed) was applied for independent comparisons: (i) between monthly MARR values of outpatients versus emergency department patients, and (ii) between internal medicine and surgery departments. Statistical significance was set at *p* < 0.05 [13].

### 2.3 Time‐Series Analysis

Given the monthly aggregated nature of the data and the temporal fluctuations observed in test volume and MARR, additional time‐series methods were employed to address potential autocorrelation, as suggested by a reviewer. First, the autocorrelation function (ACF) of the monthly MARR series was examined to detect significant lag‐dependent correlations. Second, to model the relationship between the monthly number of test requests (independent variable) and MARR (dependent variable) while accounting for any autocorrelation, an ARIMA (1,0,0) model (i.e., a regression model with an AR [1] error term) was fitted. This model was selected as the simplest parsimonious structure capable of accounting for first‑order autocorrelation, based on inspection of the autocorrelation function (ACF), which exhibited a significant spike at lag 1 and a sharp cutoff thereafter. Third, to formally compare MARR before and after the emergence of new SARS‐CoV‐2 variants, the 12‐month study period was divided into two phases: months 1–6 (April–September 2021, pre‐variant surge) and months 7–12 (October 2021–March 2022, variant‐dominant period). The Mann–Whitney U test was used to compare median MARR between these two periods. All reported p‐values are two‐sided, and no adjustment for multiple comparisons was applied because these analyses were exploratory.

The study was prepared in accordance with the Strengthening the Reporting of Observational Studies in Epidemiology (STROBE) guidelines.

## 3 Results

Over the course of 1 year, approximately four million laboratory data points—including test results for outpatients, emergency cases, and inpatients—were retrieved from Sina Hospital's HIS system. Outpatient departments accounted for 224,901 of these results, while the emergency department (ED) accounted for 378,150.

### 3.1 Comparison between Emergency and Outpatient Departments

For outpatient orders, the overall MARR was 27.0% (95% CI: 26.9% – 27.1%), whereas for ED requisitions it was 30.0% (95% CI: 29.8% – 30.2%). Although ED patients had a higher MARR, this difference was not statistically significant (Mann‐Whitney U, *p* = 0.9).

**Figure 1:**
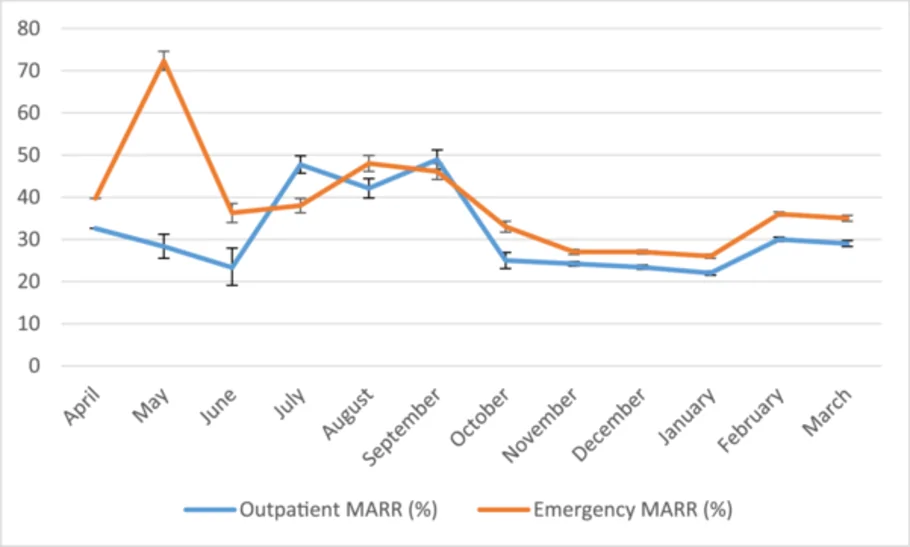
Monthly MARR score of tests ordered in outpatient and emergency department (Orange: ED, Blue: outpatient). Error bars represent 95% confidence intervals.

Examining the monthly pattern of MARR (Figure 1), the two settings showed similar fluctuations throughout the year, with increases and decreases occurring almost simultaneously, although in most months the ED MARR was higher than that of outpatients.

### 3.2 MARR of Individual Tests

**Figure 2:**
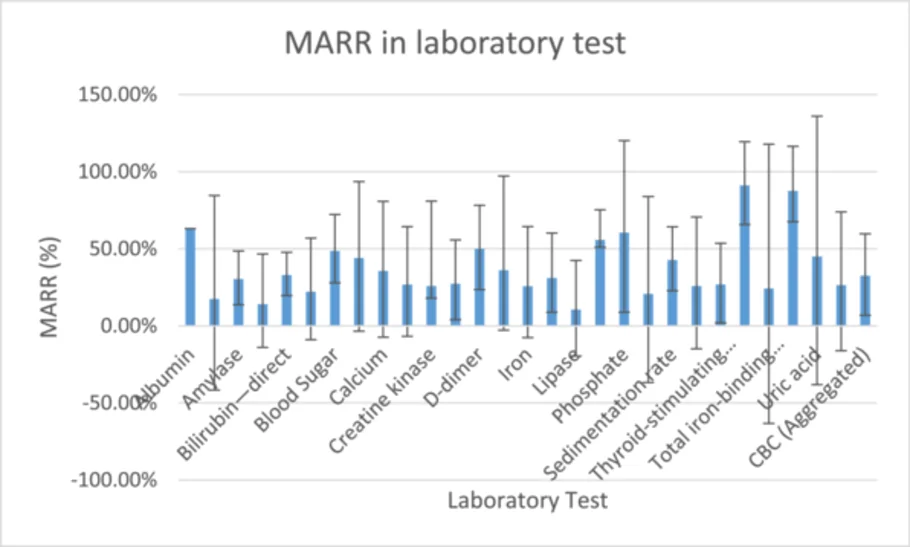
MARR score of each laboratory test included for analysis. Error bars represent 95% confidence intervals.

To assess the contribution of specific tests to the overall MARR, we identified the tests with the highest and lowest MARR scores. Among the 30 tests ordered during the study period, free T4 had the highest MARR (91%) and lipase the lowest (10%). Figure 2 displays the individual MARR scores for all 30 tests.

### 3.3 Comparison Between Internal Medicine and Surgery Departments

Separate MARR scores were calculated for internal medicine and surgical units. Internal medicine had a MARR of 30% (95% CI: 29.8%–30.2%) and surgery had a MARR of 29% (95% CI: 28.8%–29.2%), with no statistically significant difference (Mann‐Whitney U, *p* > 0.05).

### 3.4 Time‐Series Analysis of Monthly Test Volume and MARR

**Figure 3:**
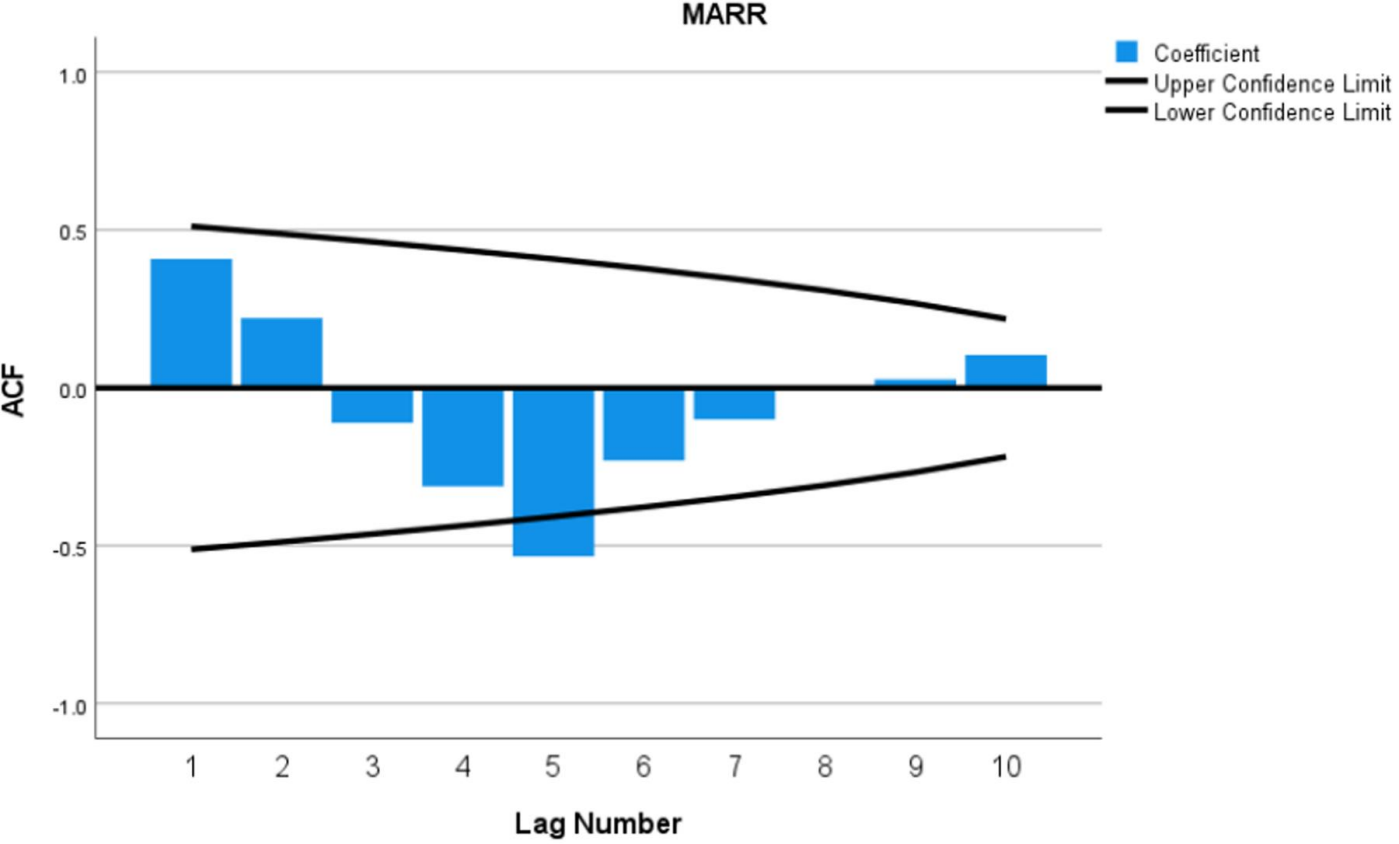
Autocorrelation function (ACF) of monthly MARR values.

The monthly number of test requests and corresponding MARR values are shown in Table 2. Visual inspection reveals wide fluctuations, with a marked increase in test volume from October 2021 onward. To formally examine the relationship between test count and MARR while accounting for potential autocorrelation, we performed an ARIMA(1,0,0) regression. The model yielded a negative coefficient for test count (estimate = –6.7 × 10^−5^, *p* = 0.114), (Table 3) indicating an inverse association that did not reach statistical significance. The autocorrelation function (ACF) of MARR showed no significant autocorrelation at lag 1 (*r* = 0.408, *p* = 0.110) but a significant negative autocorrelation at lag 5 (*r* = –0.534, *p* = 0.029), suggesting a possible periodic pattern every 5 months (Figure 3).

**Table 2:** Monthly number of tests requested and their corresponding MARR scores.

Months	April 2021	May 2021	June 2021	July 2021	Aug 2021	Sep 2021	Oct 2021	Nov 2021	Dec 2021	Jan 2022	Feb 2022	March 2022
N. of tests	7606	6172	8847	10,300	10,800	13,698	103,376	93,784	97,643	86,329	86,329	83,884
N. of abnormal results	1552	2487	3149	4243	4940	6258	30169	25471	25691	22590	21810	26484
MARR	20%	40%	35%	41%	45%	45%	29%	27%	26%	26%	25%	31%
95% CI	(19.5% – 21.3%)	(39.1% – 41.5%)	(34.6% – 36.6%)	(40.2% – 42.2%)	(44.8% – 46.7%)	(44.8% – 46.5%)	(28.9% – 29.5%)	(26.9% – 27.5%)	(26.0% – 26.6%)	(25.9% – 26.5%)	(25.0% – 25.6%)	(31.3% – 31.9%)

**Table 3:** Results of time‐series analysis and period comparison.

Analysis	Parameter/Comparison	Estimate/Value	95% CI or IQR	*p* value
ARIMA(1,0,0) model	Coefficient for test count	–6.7 × 10^−5^	(–1.5 × 10^−4^ to 1.6 × 10^−5^)	0.114
	AR [1] coefficient	0.300	(–0.37 to 0.97)	0.401
	Constant	37.96	(29.1 to 46.8)	< 0.001
Mann‐Whitney U	MARR months 1–6 (pre‐variant)	Median 41%	IQR: 35% to 45%	
	MARR months 7–12 (variant period)	Median 27%	IQR: 26% to 29%	
	Difference	–14%		0.065

It should be noted that the time‐series analysis is based on only 12 monthly observations. Consequently, the statistical power is limited, and the estimates (including the ARIMA coefficient and the ACF) may lack stability. These findings should therefore be interpreted with caution and considered preliminary until validated with a longer data series.

### 3.5 Comparison of MARR Before and After Emergence of New Viral Variants

To evaluate the impact of the COVID‐19 pandemic wave caused by new variants, we compared MARR between months 1–6 (April–September 2021, pre‐variant surge) and months 7–12 (October 2021–March 2022, variant‐dominant period). The median MARR decreased from 41% (IQR: 35–45) in the first period to 27% (IQR: 26–29) in the second period. This difference was borderline significant (Mann–Whitney U = 6.0, exact *p* = 0.065) (Table 3).

## 4 Discussion

**A group of researchers** out of Canada found through a number of studies [14] that physicians who order excessive testing can cause an increase in the number of abnormal test results and therefore result in false positive test results. These false positive results can lead to more unnecessary stress for patients and increased expenses for the healthcare system. When testing is requested on patients who show no signs of illness, the likelihood of a false positive result is greatly increased. Consequently, the development of the MARR metric was created to better quantify how appropriate testing is.

In order to assess doctors’ laboratory test ordering patterns with the goal of cost reduction and worldwide benchmarking, the research compared the MARR in outpatient and inpatient care settings as well as across several specialty hospital departments. A total of 623,051 tests were examined in a single year. There was no discernible difference between the two settings when the MARR was computed at 27% in the outpatient clinic and 30% in the emergency department after the identification of aberrant patients in both settings.

Ngo et al. [4]. discovered that the use of laboratory tests varied depending on the type of medical service, with almost all inpatients, roughly half of emergency department patients, and nearly one‐third of outpatients obtaining laboratory tests during their medical visits, even though the number of tests ordered in emergency settings is higher than in outpatient clinics. A MARR that is not substantially different from that seen in outpatient practice is the consequence of patients in inpatient facilities usually presenting with more severe medical issues, which increases the risk of aberrant outcomes in this group.

The number of tests ordered and the abnormal test rate were shown to be strongly correlated by Naugler et al. [14]. The incidence of aberrant findings declined when additional tests were added to the same request, as was to be anticipated. The abnormal incidence for requisitions with more than nine tests was just marginally higher than that of participants in good health. A research on the MARR of tests requested in Calgary's emergency rooms between 2014 and 2019 was carried out by Enwere et al. [15]. The number of tests conducted per requisition and the MARR showed a statistically significant negative connection, with the MARR falling 1.9% for each extra test requested. In our study, an ARIMA(1,0,0) time‐series model also showed a negative coefficient for the association between monthly test volume and MARR (estimate = –6.7×10^−5^), but this relationship did not reach statistical significance (*p* = 0.114). Monthly comparisons of MARR for the year 2021 indicated that MARR was higher during the spring and summer months compared to lower MARR values in the fall and winter months. Monthly comparisons were chosen because there were large increases in the volume of Covid19 testing during these periods, and they allow for an exploratory analysis of how these increases affected the overall selectivity of testing. The increasing volume of Covid19 patients in hospitals and outpatient settings during the same time period probably resulted in increased routine requisitions from physicians [16].

While looking at the connection between the MARR score and how many tests are ordered for each patient, it is important to consider how much we can actually use those abnormal lab tests. HOUBEN et al. [17]. have shown that for patients with a low chance for the condition being tested, there are many instances of abnormal laboratory results for reassurance or for exclusion of disease. A lot of this can be attributed to how abnormal these tests were; that is, most were very slight in nature. So, while we may see no substantial difference in the scores of the MARR for patients who have undergone extensive testing, the way in which these patients react to their abnormal lab results will likely be similar to those who have normal results.

Different studies provide varying MARR scores for different lab tests. In the study conducted by Enwere et al. [15]., the highest overall per‐test MARR score was BNP ‐ with a score of 80.5% ‐ while the lowest score was glucose ‐ with a score of 7.9%. In contrast, in the study conducted by Houben et al. [17]., creatinine levels had the highest proportion of abnormal results (61%); whereas average corpuscular volume (MCV) had the lowest proportion (6%). In a comparison of MARR scores for 30 tests performed at Sina Tabriz Hospital, the Free T4 test had the highest MARR score, indicating that physicians were more likely to order this test in selective situations; however, the Lipase test had the lowest MARR score, suggesting that this test was requested frequently in the emergency department for acute illnesses.

Different test selections, depending on where they are being tested, have the potential to affect MARR scores because they are influenced by such things as: geographic area; following the recommendations of initiatives like the Choosing Wisely campaign; and the medical judgment and experience of the individual medical provider. In addition, many of the tests evaluated in this study are typically performed as part of a standard panel when testing for specific disease processes, and therefore have the potential to affect the overall MARR. The findings also indicate that the tests included in this evaluation tend to be relatively high‐volume, inexpensive, and accessible, which could allow for over‐utilization or over‐ordering of these tests versus other more expensive or specialized tests. Brack et al. [18] have shown that on average, after 5 years of promotion, physician awareness of choosing wisely increased MARR in Calgary from 8.1% to 9.0%, which correlated with their increased use of MARR. In addition, MARR has been associated with the physician's level of clinical experience [15].

Studies have shown that laboratory resources came under strain due to the COVID‐19 pandemic [19] with reports of a 10% increase in the average number of tests ordered for this period of time when compared to what would normally be ordered (11% increase). The reported increase in tests ordered correlates to an increased demand for all routine tests that are used for the management and diagnosis of COVID‐19 and a reduced number of tests ordered for lower‐level or less important tests during this period of time.

In the second half of 2021 (July to December 2021), we observed a substantial decrease in MARR when new mutant variants of COVID‐19 entered the country. The number of physician orders for CBC, CRP, and ESR testing increased approximately 13‐fold (from 7,606 in April to 103,376 in October, Table 2). In addition to the significant increase in the number of physician orders for testing, the increase is also due, in part, to the number of people infected with COVID‐19 and being tested as being suspected of having the virus, as demonstrated by the results of the tests.

Vaccination campaigns in Iran which began in May 2021 [20] were likely key drivers of decreased levels of concern about COVID‐19 in the general population, hence an increase in unnecessary referrals and testing requests. The combination of these factors was likely responsible for a further drop in MARR over this period. Using a Mann‐Whitney U test to compare MARR between months 1–6 (pre‐variant) and months 7–12 (variant period), the median MARR decreased from 41% to 27%, with a borderline significant difference (*p* = 0.065). This suggests a trend toward reduced test selectivity during the variant wave, although the statistical evidence is not conclusive at the conventional α = 0.05 level.

Overall, there was no statistical difference between the MARRs of the Internal Medicine and Surgery Departments. The similarity in MARRs may be attributed to the American Society for Clinical Pathology's recommendation to limit routine preoperative testing to low‐risk surgeries unless specifically indicated [21]. Therefore, it was expected that both departments would have a similar rate of utilization of laboratory services for their admitted patients, factoring in the potential influence of COVID‐19.

There are a number of limitations to take into account. First, the effect of physician experience and specialization on MARR could not be directly evaluated in this study, as we lacked individual‐level data on years of practice or training. The absence of a significant difference between clinical settings or specialties does not constitute evidence that physician experience has no impact; such an inference would require a dedicated study with provider‐level variables. Second, marginally abnormal findings may have limited diagnostic or therapeutic value, since the definition of “abnormal” was based on the reference ranges of a single facility. Third, MARR serves only as an indirect proxy for appropriateness. An abnormal result does not necessarily indicate a clinically meaningful finding, and a normal result may still be appropriate in certain contexts. Without linking each test to its clinical indication, pre‐test probability, or patient outcome, conclusions about over‐ or under‐utilization remain inferential. Fourth, the aggregation of test components may obscure variability in test performance, and the inclusion of mildly abnormal values may inflate MARR estimates without reflecting actionable abnormalities. Finally, the exploratory time‐series analyses were limited by the small number of monthly time points (n = 12), which precluded more complex models.

## 5 Conclusion

From the results of this investigation, an inverse temporal pattern was observed between MARR and test volume, although the association was not statistically significant after accounting for autocorrelation (*p* = 0.114). No significant differences in MARR were found between emergency and outpatient settings or between internal medicine and surgery departments. This study found that the coronavirus pandemic had a considerable effect on test ordering patterns and the associated MARR, as test requests increased twelvefold in the autumn and winter while MARR decreased by the same amount. During the period of new viral variant emergence, test requests increased approximately 13‐fold (from 7,606 to 103,376 per month) and MARR showed a borderline significant decline (from median 41% to 27%, exact *p* = 0.065). Therefore, we suggest using MARR as an additional metric to evaluate the relative selectivity of test ordering by certain doctors. This may include calculating provider‐specific MARRs and providing feedback for audit and educational purposes. However, MARR should not be interpreted as a direct measure of appropriateness or physician experience without case‐mix adjustment and individual‐level clinical data.

## Author Contributions

**Parastou Gorovanchi:** supervision, data curation, formal analysis, resources. **Parham Panahi:** methodology, software. **Zahra Farrokhi:** writing – review and editing, writing – original draft. **Rasoul Hossein Zadeh:** data curation. **Maryam Baradaran‐Binazir:** resources, software. **Rasoul Estakhri:** conceptualization, supervision, resources.

## Funding

The authors have nothing to report.

## Ethics Statement

The study protocol was approved by the Ethics Committee of Tabriz University of Medical Sciences (Approval No. 70222). Due to the retrospective nature of the study and the use of anonymized data, individual informed consent was waived by the ethics committee.

## Conflicts of Interest

The authors declare no conflicts of interest.

## Transparency Statement

Parastou Gorovanchi affirms that this manuscript is an honest, accurate, and transparent account of the study being reported; that no important aspects of the study have been omitted; and that any discrepancies from the study as planned (if any) have been explained.

## Data Availability

The data supporting the findings of this study are available from the corresponding author upon reasonable request, subject to institutional ethical restrictions. The data are not publicly available to protect patient privacy.
